# Simultaneous Genome Sequencing of *Prosthecochloris ethylica* and *Desulfuromonas* *acetoxidans* within a Syntrophic Mixture Reveals Unique Pili and Protein Interactions

**DOI:** 10.3390/microorganisms8121939

**Published:** 2020-12-07

**Authors:** John A. Kyndt, Jozef J. Van Beeumen, Terry E. Meyer

**Affiliations:** 1 College of Science and Technology, Bellevue University, Bellevue, NE 68005, USA; 2 Department of Biochemistry and Microbiology, Ghent University, 9000 Gent, Belgium; jozef.vanbeeumen@ugent.be; 3 Department of Chemistry and Biochemistry, University of Arizona, Tucson, AZ 85721, USA; temeyer@email.arizona.edu

**Keywords:** green sulfur bacteria, syntrophy, e-pili, adhesion protein, photosynthetic symbionts, large multiheme cytochrome, metagenomic binning

## Abstract

Strains of *Chloropseudomonas ethylica*, 2-K, N2, and N3 are known to be composed of a syntrophic mixture of a green sulfur bacterium and a sulfur-reducing colorless component. Upon sequence analysis, the green sulfur photosynthetic bacterial component of strain N3 was dominant and was readily sequenced, but the less abundant sulfur-reducing bacterial component was apparent only when analyzed by metagenomic binning. Whole-genome comparison showed that the green bacterium belonged to the genus *Prosthecochloris* and apparently was a species for which there was no genome sequence on file. For comparison, we also sequenced the genome of *Prosthecochloris* sp. DSM 1685, which had previously been isolated from the 2-K mixture in pure culture and have shown that all three *Prosthecochloris* genomes belong to a new species, which we propose to be named *Prosthecochloris ethylica* comb. nov. Whole genomes were also sequenced for the isolated *Desulfuromonas* strains DSM 1675 (from strain 2-K) and DSM 1676 (from strain N2) and shown to be nearly identical to the genome found in the N3 mixture. The genome of the green sulfur bacterium contains large genes for agglutination proteins, similar to the ones proposed to be involved in larger photosynthetic consortia of *Chlorochromatium aggregatum*. In addition, we also identified several unique “tight adhesion (tad)” pili genes that are presumably involved in the formation of cell–cell interactions. The colorless component, on the other hand, contained a unique large multiheme cytochrome C and unique genes for e-pili (geopilin) formation, genetically clustered with a conserved ferredoxin gene, which are all expected to play an electron transfer role in the closed sulfur cycle in the syntrophic mixture. The findings from the simultaneous genome sequencing of the components of *Cp. ethylica* have implications for the phenomenon of direct interspecies interactions and coupled electron transfer in photosynthetic symbionts. The mechanisms for such interactions appear to be more common in the environment than originally anticipated.

## 1. Introduction

*Chloropseudomonas ethylica* was originally described as a motile green sulfur bacterium capable of utilizing ethanol [1]. However, no pure cultures of green sulfur bacteria were previously known that would utilize ethanol or that were motile. Strains of *Cp. ethylica* were isolated from the Krujalnik Estuary, near Odesa, Russia (strain 2-K) and from Lake Saksky in Crimea, Russia [2], which in hindsight is a remarkable coincidence considering the approximately 200-mile distance between these habitats. However, doubts were raised about the purity of the strains when two different cell morphologies were observed in growing cultures [2,3]. Subsequently, the colorless, motile, ethanol-oxidizing, and sulfur-reducing bacterium *Desulfuromonas acetoxidans* strains DSM 1675 and DSM 1676 were isolated from either the 2-K or the N2 syntrophic mixtures [4]. A pure culture of *Ds. acetoxidans* DSM 684^T^ was also isolated from South Orkney Island, Antarctica [4]. The green component was either described as a species of *Chlorobium* [2] or *Prosthecochloris* [3]. The amino acid sequences of cytochrome C-555, alternatively known as c5, from the *Cp. ethylica* 2-K mixture was clearly related to those of *Prosthecochloris* species and not to *Chlorobium* [5], whereas the cytochrome C-551.5, alternatively known as c_7_ [6] came from the *Desulfuromonas* component [7]. It is unclear whether there is a specificity to the connection between the two species in the *Cp. ethylica* mixture. Agar shake cultures with ethanol, sulfide, and bicarbonate show large green colonies (faster-growing when involved in sulfur cycling) and small green colonies. The large ones were verified to be in mixed culture and the smaller ones as a pure culture [8] and our unpublished observations.

Mutualistic relationships that involve close cell–cell interactions are most studied between bacterial and eukaryotic interactions, e.g., between nitrogen-fixing *Rhizobium* species and legumes, or bacterial pathogens and eukaryotic hosts. Symbiotic interactions amongst archaea and bacteria can be found in microbial mats where nutrient exchange and waste removal roles are crucial, in anaerobic methane-oxidizing communities of marine environments or in human digestive systems [9,10,11,12,13]. These interactions have only recently been studied in more detail and appear to be more common than historically expected. Larger bacterial and archaeal consortia that are formed through cell–cell interactions of two or more microorganisms have been observed to form a high degree of interdependence between taxonomically unrelated species [14,15,16]. Phototrophic consortia of this sort were first reported over a century ago [17]. The nature of the *Cp. ethylica* syntrophy appears to be centered around a closed sulfur cycle [18], similar to what has been shown in syntrophic cocultures of *Chlorobium phaeovibrioides* and *Desulfuromonas acetoxidans*, where acetate is oxidized by *Ds. acetoxidans*, with sulfur as an electron acceptor [19]. The process leads to the recycling of the sulfide that can then be used for anoxygenic photosynthesis by *Cb. phaeovibrioides*. Although these syntrophic cocultures appear to be more widespread than commonly expected and the nutrient cycle and mutual benefits are clear in some cases, very little is known about the physical interaction and formation of these cell–cell interactions and the specific components of the electron chemistry involved to establish such a mutually beneficial nutrient cycle. Studies with larger phototrophic consortia, such as *Chlorochromatium aggregatum*, have shown that the green sulfur bacteria involved in this complex are likely preadapted to a symbiotic lifestyle, and specific ultrastructures (periplasmic extruding tubules) can be formed between the central bacterium and the epibiont [14,20]. *Cp. ethylica* was not described to form larger aggregates and appears to form a simpler model of syntrophy. However, the formation of such larger consortia, even in *Chlorochromatium aggregatum*, is dependent on the cultivation strategy. To gain further insight into the possible physical interactions and the electron transfer mechanism involved in *Cp. ethylica*, we set out to determine the genomes of the syntrophic mixtures. 

We now report the simultaneous determination of the genome sequences of the green and colorless components of the *Cp. ethylica* N3 mixture. Although the genome sequence of *Desulfuromonas acetoxidans* DSM 684^T^ was previously determined [21], we have now also established the genome sequences of the *Desulfuromonas* strains 2-K (DSM 1675) and N2 (DSM 1676), previously isolated from two of the *Cp. ethylica* mixtures. We attempted to simultaneously determine the sequences of both organisms from strain N2, but obtained only the genome belonging to the green sulfur bacterial genome, likely due to slightly different culture conditions that may have led to a lower abundance of the colorless component in the mixture.

## 2. Materials and Methods 

### 2.1. Cultures and DNA Preparation

Cultures of *Chloropseudomonas ethylica* N3 and N2 were originally obtained by one of us (Terry E. Meyer) directly from the laboratory of E.N. Kondrat’eva, and had been grown in our laboratory over the years and kept in a lyophilized state. Cultures were grown according to [8] and harvested by centrifugation. DNA extracted from decades-old frozen cultures of the N3 and N2 syntrophic mixtures were the source for the genomic analysis presented here. Genomic DNA was extracted using the GeneJET DNA purification kit (Thermo Scientific, Waltham, MA, USA). The quantity and purity of DNA, determined using Qubit and Nanodrop instruments, showed an A260/280 ratio of 1.75.

Genomic DNA for *Prosthecochloris* sp. DSM 1685, *Desulfuromonas acetoxidans* DSM 1675 and DSM 1676 was obtained from DSMZ (Deutsche Sammlung von Mikroorganismen und Zellkulturen, GmbH). The quantity and purity of DNA, determined using Qubit and Nanodrop instruments, showed A260/280 ratios between 1.8 and 1.9.

### 2.2. DNA Sequencing, Assembly, and Annotation

The DNA libraries were prepared with the Nextera DNA Flex Library Prep Kit (Illumina, Inc., San Diego, CA, USA). All five genomes were sequenced, using 500 μL of a 1.8 pM library, with an Illumina MiniSeq instrument, using paired-end sequencing (2 × 150 bp). Quality control of the reads was performed using FASTQC within BaseSpace (Illumina, version 1.0.0), using a k-mer size of 5 and contamination filtering. The data for each of the genomes of *Chloropseudomonas ethylica* N3 and N2, *Prosthecochloris* sp. DSM 1685, and *Desulfuromonas acetoxidans* DSM 1675 and DSM 1676 was assembled *de novo* using Unicycler within PATRIC (Pathosystems Resource Integration Center) [22]. The genome sequences were annotated using RAST (Rapid Annotations using Subsystem Technology; version 2.0) [23].

Average percentage nucleotide identity (ANIb) between the whole genomes was calculated using JSpecies [24]. A whole-genome-based phylogenetic tree was generated applying the CodonTree method within PATRIC [22], which used PGFams as homology groups. Moreover, 519 PGFams were found among these selected genomes using the CodonTree analysis to construct the *Prosthecochloris* tree, while 598 unique PGFams were identified for the *Desulfuromonas* tree. The aligned proteins and coding DNA from single-copy genes were used for RAxML analysis [25,26] for the trees. The support values for the phylogenetic tree were generated using 100 rounds of the “Rapid bootstrapping” option of RaxML. Tree visualization was performed with iTOL [27].

This Whole-Genome Shotgun project has been deposited at DDBJ/ENA/GenBank, and the accession numbers for all of the sequenced genomes are listed in Table 1.

### 2.3. Metagenomic Binning

The sequencing reads of *Cp. ethylica* N3 were used to perform a metagenomic binning using the Metagenomic Binning service within PATRIC [22]. Paired-end reads were used as input, and default parameters were used. Sets of contig bins were constructed, with hits against contigs that have less than fourfold coverage or are less than 400 bp in length being removed. The contig pool was split into bins using reference genomes. Quality control of each bin was performed using checkM [28]. Each bin was automatically annotated using RAST within PATRIC [22], and consistency checks of the annotation were performed, producing a coarse score (percentage of roles that are correctly present or absent) and a fine score (percentage of roles that are correctly absent or present in the correct number). Identified genomes were ranked based on their coarse score, fine score, and completeness. 

### 2.4. Synteny Analysis

For synteny analysis, comparative genome regions were generated using global PATRIC PGFam families to determine a set of genes that match a focus gene. All genomes were used in the search and compared to the reference genome. The gene set was compared to the focus gene using BLAST and sorted by BLAST scores within PATRIC [22]. The *Prosthecochloris ethylica* N3 TadZ/CpaE (PGFam_00109911) and agglutination protein (PGFam_02064367) were used as focus genes to analyze synteny of the Tad pili and adhesion protein gene clusters, respectively. For the *Desulfuromonas acetoxidans* synteny comparisons, the Type IV major assembly protein PilA (PGFam_00056426) and cytochrome C-551.5 (PGFam_10701576) were used as focus genes. 

## 3. Results

The results of the genome sequences that were determined in this work, i.e., N2 and N3 syntrophic mixtures, those of the pure cultures DSM 1675 and DSM 1676 (isolated from the N2 and 2-K mixtures), and the pure culture DSM 1685 (from the 2-K mixture) are shown in Table 1. Knowing that *Cp. ethylica* N2 and N3 consists of a mixture of two species, we attempted to separate the raw genome data by metagenomic binning. In the case of *Cp. ethylica* N3, we obtained two valuable bins, one genome related to *Prosthecochloris* sp. (97.0% coarse consistency and 96.7% fine consistency), the other to *Desulfuromonas acetoxidans* (98.4% coarse consistency and 97.3% fine consistency), both with 100 % completeness (included in Table 1). From the *Cp. ethylica* N2 data, we only obtained the genome of the *Prosthecochloris* sp. 

### 3.1. Prosthecochloris Genome from the Green Sulfur Component

Average nucleotide identity (ANIb) comparison showed that the genome sequences of the green component of the N2 and N3 mixtures were virtually the same as the one isolated from the 2-K mixture (DSM 1685) and both were similar to those of *Prosthecochloris* species, with an average nucleotide identity (ANI) of 75% to the nearest, previously determined, strain HL130 (Table 2). The ANI values of all the other *Prosthecochloris* species are well below 95%, which is the arbitrary cutoff value for species differentiation [24]. It is, therefore, likely that the genome sequence of the green component of the N2, N3, and 2-K mixture is from a new species, which we propose to be called *Prosthecochloris ethylica* comb. nov.

**Figure 1:**
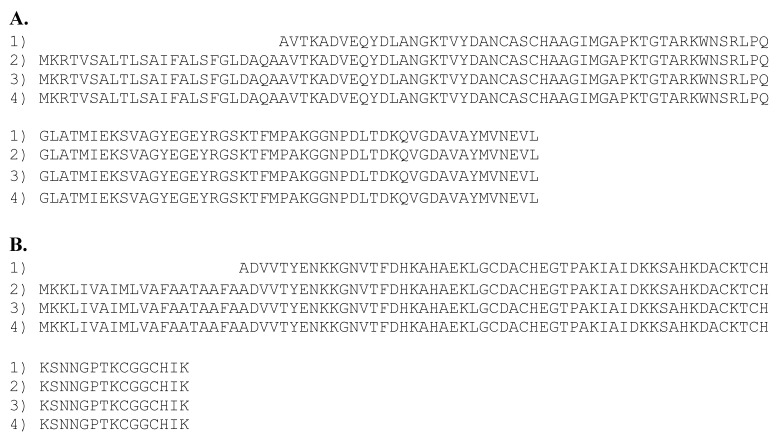
(**A**) Sequence alignment of cytochromes c5 from *Prosthecochloris*. (1) Van Beeumen et al. [5] soluble protein from 2-K mix, (2) translated genome from pure 2-K DSM 1685, (3) translated genome from N2 mix, and (4) translated genome from N3 mix. (**B**) Sequence alignment of cytochromes c-551.5 from *Desulfuromonas.* (1) Ambler [6] soluble protein from 2-K mix, (2) translated genome from pure 2-K DSM1675, (3) translated genome from pure N2 DSM1676, and (4) translated genome from N3 mix.

As expected, these genomes contained a gene for cytochrome C-555, for which the translated protein sequences of strains N2 and N3 were identical (100% identity) to that previously reported for the green component of the *Cp. ethylica* strain 2-K mixture [5], as shown in Figure 1. 

**Figure 2:**
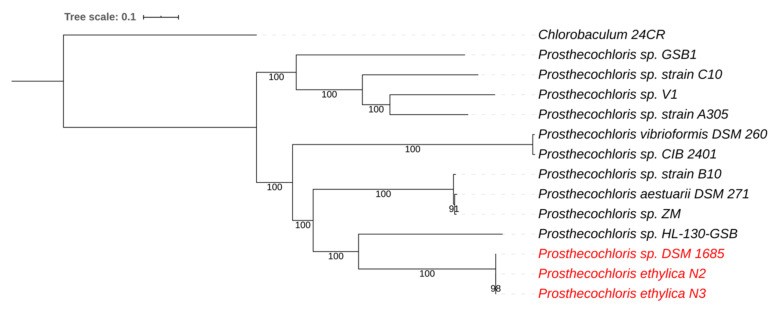
Whole-genome-based phylogenetic tree of all sequenced *Prosthecochloris* species. The phylogenetic tree was generated using the CodonTree method within PATRIC [22], which used PGFams as homology groups. The support values for the phylogenetic tree are generated using 100 rounds of the “Rapid bootstrapping” option of RaxML [25]. *Chlorobaculum* sp. 24CR was used as an outgroup [29]. The branch length tree scale is defined as the mean number of substitutions per site, which is an average across both nucleotide and amino acid changes. Species marked in red belong to the newly proposed *Prosthecochloris ethylica* comb. nov.

A whole-genome phylogenetic tree for *Prosthecochloris* placed the green component of the N2 and N3 mixtures as nearly similar to that of strain DSM 1685, isolated from the 2-K mixture (red clade in Figure 2). This is consistent with the ANI comparisons mentioned above and clearly distinguishes this clade as separate from the other sequenced *Prosthecochloris* strains, with strain HL130 as the closest relative. This further supports the proposal of a new *Prosthecochloris* species.

**Figure 3:**
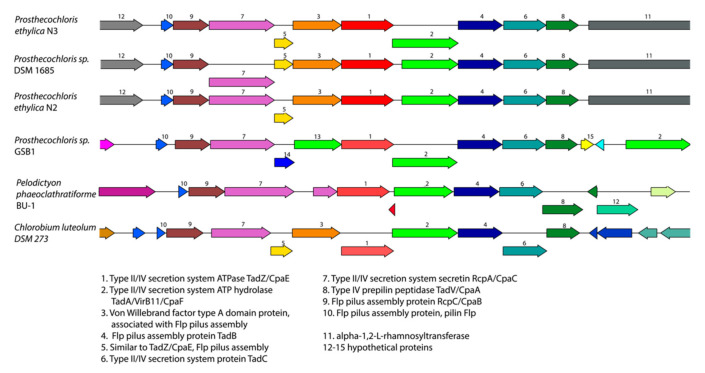
Gene cluster organization and synteny comparison of the Tad (Tight Adhesion) genes identified in the Prosthecochloris genomes. Genes are colored according to protein family (PGFam).

A search for protein families that are unique amongst the *Prosthecochloris* strains using PATRIC revealed a Tad (Tight Adhesion) pili gene cluster that is found exclusively in strains N2, N3, and DSM1685 and with lower homology in strains GSB1 and CIB2401, but appears to be absent from all other *Prosthecochloris* strains. This Tad pili gene cluster consists of at least 10 genes related to Type II/IV Flp pili assembly and secretion. The synteny of the gene cluster is preserved in all these species (Figure 3), indicating an evolutionary conservation of this cluster. The closest relatives with a similar gene cluster were found to be *Pelodictyon phaeoclathratiforme* BU-1 and *Chlorobium luteolum* DSM 273. Interestingly, *P. phaeoclathratiforme* is a brown-colored *Chlorobiaceae* that was described to form net-like colonies [30]. 

Tad pili gene clusters encode a macromolecular transport system (type II secretion system). They are present in the genomes of a wide variety of both Gram-negative and Gram-positive bacteria and are involved in close adhesion of cells within biofilm formation, colonization, and, sometimes, pathogenesis [31,32,33,34]. The long filamentous fibrils are formed by bundles of individual pilus strands, consisting of the fimbrial low-molecular-weight protein Flp [35,36,37]. Their attachment to surfaces and other cells are expected to create an optimized environment for nutrient, metal, and electron exchange between cells [31,33]. Given the presence of these conserved genes and the synteny in the *Prosthecochloris* strains isolated from the three *Cp. ethylica* mixtures, these Tad-encoded pili likely play a similar role in the syntrophic relationship of these strains by forming cell–cell interactions. 

It has been described earlier that in larger multicellular phototrophic consortia of *Chlorochromatium aggregatum*, a few large genes encode proteins that are anticipated to play an important role in the formation of close interspecies interactions [14]. This was suggested based on in silico analysis, but the exact physical role of these proteins in forming these interactions have not been described. The four involved putative ORFs showed similarities to hemagglutinins (2 ORFs), an RTX toxin and hemolysin, and were found to be some of the largest genes detected in prokaryotes and to contain multiple characteristic repeats [13,14]. When searching for large ORFs in the *Prosthecochloris* genomes that we sequenced, we found the largest ORF (coding for 2417 aa.) to be annotated as “hypothetical protein.” When performing an NCBI BLASTP search using this sequence, we found it to be homologous to an “outer membrane adhesin-like protein” from *Pelodictyon phaeoclathratiforme* and a “tandem-95 repeat” protein from *Prosthecochloris aestuarii*, albeit with low protein identity (<39%). Further comparison showed that all three of these proteins are homologous to hemagglutinin/adhesin-like proteins, similar to outer membrane adhesin proteins of the RTX toxin family, which contain numerous, internally repeated, calcium-binding domains [38]. 

**Figure 4:**
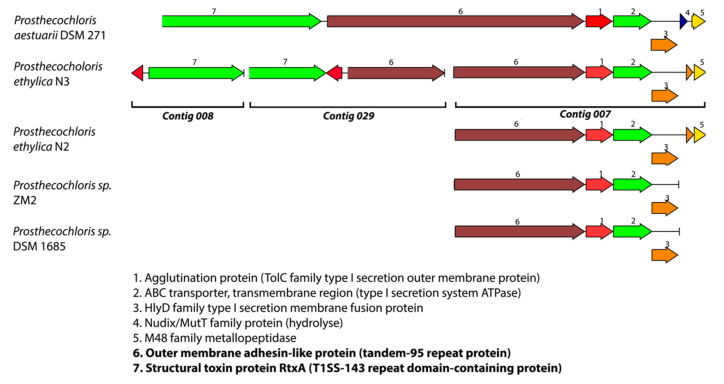
Gene cluster organization and synteny comparison of the large adhesin-like protein-encoded genes, identified in the *Prosthecochloris* N3 genome and homologues. The genes for large extracellular outer membrane adhesion protein and RtxA discussed in the text are marked in bold.

Although highly diverse in terms of structure and/or adhesives properties, outer membrane adhesins in Gram-negative bacteria are usually grouped into two main categories: the adhesins secreted through a type 1 secretion system (T1SS) and the adhesins secreted through one of the type 5 secretion systems (T5SS) [39]. The most studied of these secreted adhesins are the biofilm-associated family of proteins (Bap), which are high-molecular-weight multidomain proteins containing an N-terminal secretion signal, a core domain of highly repeated motifs, and a glycine-rich C-terminal domain. Bap family members have been shown to be involved in cell adhesion to abiotic surfaces and biofilm formation in both Gram-positive and Gram-negative bacteria (for reviews, see [40,41]). However, only a few of these proteins have been characterized experimentally. A closer look at the gene region surrounding the large *Prosthecochloris* putative adhesion protein revealed, first, that the gene is located at the end of a contig in the genomes of strains N3, N2, and DSM1685, indicating that the full size of the encoded protein might be larger than 2417 amino acids and, second, that the gene is followed by a gene encoding an agglutination protein (TolC family type I secretion outer membrane protein), an ABC transmembrane transporter (type I secretion system ATPase), and a HlyD homologous protein (type I secretion membrane fusion protein) (Figure 4). Comparison of this gene region to closely related genomes showed that *Pr. aestuarii* DSM 271 contains a complete sequence for the adhesin-like gene (encoding 4748 amino acids) and is preceded by a large protein (4983 amino acids) that is homologous to the structural toxin protein RtxA (which is a T1SS-143 repeat domain-containing protein) in a similar gene cluster. The presence of the large RtxA homologue and the adhesin-like protein in a gene cluster with other T1SS-related proteins indicates that these genes indeed encode adhesins, secreted through a type 1 secretion system. 

The occurrence of the adhesin-like gene at the end of a contig in our *Prosthecochloris* genomes is likely due to the fact that these genes contain multiple sequence repeats, which are known to be a potential challenge for next-generation sequencing-based genome assembly programs [42]. When searching the N3 genome with the adhesin and RtxA toxin-like proteins from *Pr. aestuarii* DSM 271 (using BLASTP in PATRIC), we did find partial genes (annotated as hypothetical proteins) located at the ends of two other contigs (contigs 008 and 029). These partial gene-containing contigs from strain N3 align very well with the gene cluster identified in strain DSM 271 (see Figure 4), which supports the hypothesis that the missing partial adhesin gene is due to assembly software limitations. The same was true for the 2-K (DSM1685) and N2 genomes where the RtxA homologue was also found on separate contigs. We can conclude that the *Prosthecochloris* genomes from the 2-K, N2, and N3 mixtures all contain large genes similar to the putative ORFs (hemagglutinin and RTX toxin) that were described in *C. aggregatum* to be important for the formation of close interspecies interactions [13,14]. 

### 3.2. Desulfuromonas Genome from the Colorless Sulfur-Reducing Component

**Figure 5:**
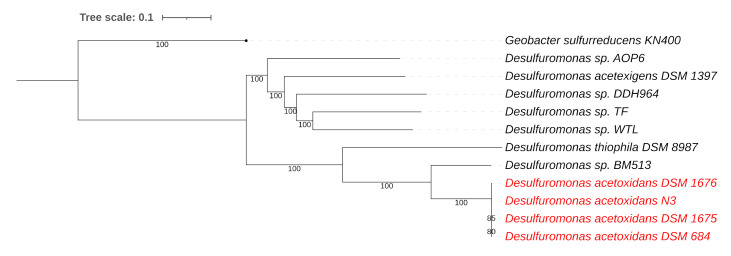
Whole-genome-based phylogenetic tree of all sequenced *Desulfuromonas* species. The phylogenetic tree was generated using the CodonTree method within PATRIC [22], which used PGFams as homology groups. The support values for the phylogenetic tree are generated using 100 rounds of the “Rapid bootstrapping” option of RaxML [25]. *Geobacter sulfurreducens* KN400 was used as an outgroup [43]. The branch length tree scale is defined as the mean number of substitutions per site, which is an average across both nucleotide and amino acid changes.

Based on ANIb comparisons (Table 3) and whole-genome phylogenetic analysis (Figure 5) of the colorless component in the N3 mixture, we can conclude that it is very similar to *Ds. acetoxidans* DSM 1675 and DSM 1676, previously isolated from the mixtures 2-K and N2, and that they are nearly the same as the type strain DSM 684.

**Figure 6:**
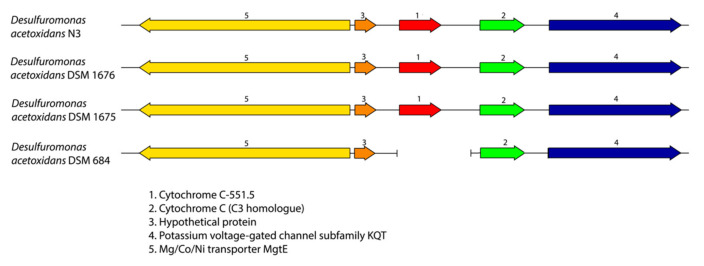
Gene organization comparison around the cytochrome *c*-551.5 gene in *Desulfuromonas* genomes. Genes are colored according to protein family (PGFam).

The *Cp. ethylica* 2-K cytochrome C-551.5 protein [6] was identical to that of the translated gene from the *Desulfuromonas* component of the N3 mixture and from the DSM 1675 and DSM 1676 pure culture, as shown in Figure 1B, but was apparently absent from the type strain DSM 684. The C-551.5 gene synteny is conserved in the N3 mixture, DSM 1675, and DSM 1676 genomes (Figure 6), and the gene is surrounded in all of the strains by a gene for cytochrome C (PGFam_04122568; located downstream) and a Mg/Co/Ni transporter MgtE (PGFam_04560429; upstream and antisense). These surrounding genes are both present in strain DSM 684; however, they are each located near the end of separate contigs (Figure 6). It is, therefore, possible that the C-551.5 gene was lost during assembly of the DSM 684 genome. Further studies will be needed to conclude the presence of C-551.5 in the type strain.

The genomes of *Desulfuromonas* strains DSM 1675, DSM 1676, DSM 684^T^, and the genome isolated from the N3 mixture contain many genes from unique protein families (PGFams identified in PATRIC) that are apparently not found in the other sequenced *Desulfuromonas* strains. At least 18 unique PGFams were related to the synthesis of Type IV pili of two different classes. Eight of them encoded mannose-sensitive hemagglutinin (MSHA) type pili, and the other 10 encode the elements of a different Type IV pili group (Table 4). Type IV pili are found on the surface of a variety of Gram-negative bacteria [44] and have been demonstrated to be important as host colonization factors, bacteriophage receptors, mediators of DNA transfer and, more recently, also as electron transfer factors over longer distances (so called e-pili) [45,46,47]. 

The type IV major pilin assembly protein PilA found exclusively in the four *Desulfuromonas* strains is significantly larger (314 residues) than the common PilA homologue found in many other bacteria (only ~60–70 aa.). The typical shorter PilA homologue was also found in all of the sequenced *Desulfuromonas* strains. This larger PilA protein (PGFam_00056426) contains a transmembrane region (from ~residues 70–160), and a BLASTP search revealed a geopilin domain membrane protein (247 aa.; PGFam_10038571) from *Pelobacter carbinolicus* DSM 2380 as the closest relative (49% protein identity and 67% similarity). Geopilin proteins have been implicated in direct interspecies electron transfer (e-pili) within syntrophic aggregates [48,49,50] and have also been shown to enhance current production in fuel cells [51]. 

**Figure 7:**
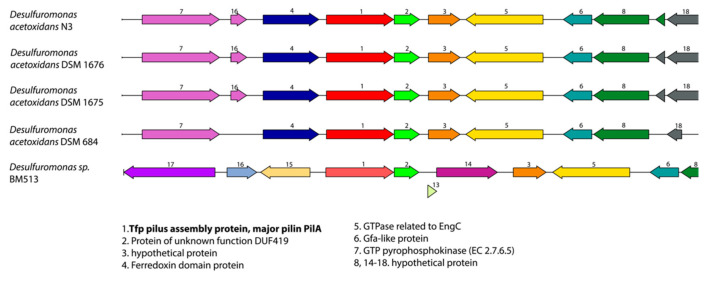
Gene organization comparison around the geopilin-PilA pilus assembly protein in *Desulfuromonas* genomes. Genes are colored according to protein family (PGFam). The gene for the large major pilin protein PilA, discussed in the text, is marked in bold.

The synteny of the geopilin-PilA protein is conserved in all four of our sequenced *Desulfuromonas* strains (Figure 7). The gene is preceded by a ferredoxin domain protein (PGFam_00004340) and followed by DUF419 (a protein of unknown function; PGFam_00038332), which are both also unique to these four *Desulfuromonas* strains. Ferredoxins are small proteins containing iron-sulfur clusters and function as biological “capacitors” that can accept or discharge electrons and are involved in electron transfer reactions in many organisms (for review see [52]). The presence of the ferredoxin protein directly upstream of the geopilin, which is proposed to be an electron-transfer pili (e-pili), is certainly intriguing and points towards a functional coupling of these proteins. In addition, unique PGFams for PilZ, PilX, PilV, PilP, and PilW, as well as FimT biogenesis proteins and the Pilin glycosylation protein PglB were found at other locations in our selected genomes. Using BLASTP (within PATRIC), we were able to identify one other *Desulfuromonas* strain, BM513, which contains a distant homologue of the larger PilA protein (66% sequence identity); however, the gene synteny is less conserved (Figure 7). This latter genome was assembled from a metagenomic sample of an environmental isolate, and nothing is currently known about potential symbiotic relationships of this strain. The presence of this larger geopilin-PilA homologue and several of the type IV pili biogenesis proteins indicates that the *Desulfuromonas* strains N3, N2, 2-K, and possibly the environmental strain BM513, are able to produce a unique set of type IV pili (e-pili) that could play a role in syntrophy and electron transfer. 

The mannose-sensitive hemagglutinin (MSHA; Table 4) is likewise a member of the family of type IV pili. While the exact function of MSHA is unknown, studies have shown that *msh*A mutants of *Vibrio cholerae* are unable to produce biofilms on abiotic surfaces, and these pili might have an environmental role of survival outside the host [53]. Two homologues of MshA were found to be present in DSM 1675, DSM 1676, DSM 684, and the N3 strain, in addition to homologues for MshD, I, J, K, N, and P, which are essential for MSHA pili biosynthesis (Table 4). Although these Msh PGFams were not found by PATRIC in the other *Desulfuromonas* strains, we also identified, by performing a BLASTP search within all *Desulfuromonas* strains in PATRIC, more distant homologues (<40% aa. identity) of MshA, and found the same conserved gene cluster in the genomes of *Desulfuromonas* sp. AOP6, BM513, and *Ds. thiophila*. This indicates that the *msh* pili gene cluster might be more widespread amongst the *Desulfuromonas* species. 

Besides these pili genes, the *Desulfuromonas* genomes also contain genes for several flagellar proteins, including the flagellar assembly protein FliH, flagellar basal body P-ring formation protein FlgA, the basal body rod protein FlgF, flagellar protein FlgJ (2 copies), and a flagellar regulatory protein FleQ. These were initially found as unique families by PATRIC in strains N3, DSM 1675, DSM 1676, and DSM 684. However, when performing a BLAST search, we found homologues of them (<45% protein identity) in several of the other strains, e.g., DSM1397, AOP6, and *Ds. thiophila*. This is consistent with the earlier observations that the *Desulfuromonas acetoxidans* species in the syntrophic mixtures are motile and able to produce functional flagella [8]. 

### 3.3. Comparison of the Geobacter sulfurreducens Genomes to the Desulfuromonas Genomes

**Figure 8:**
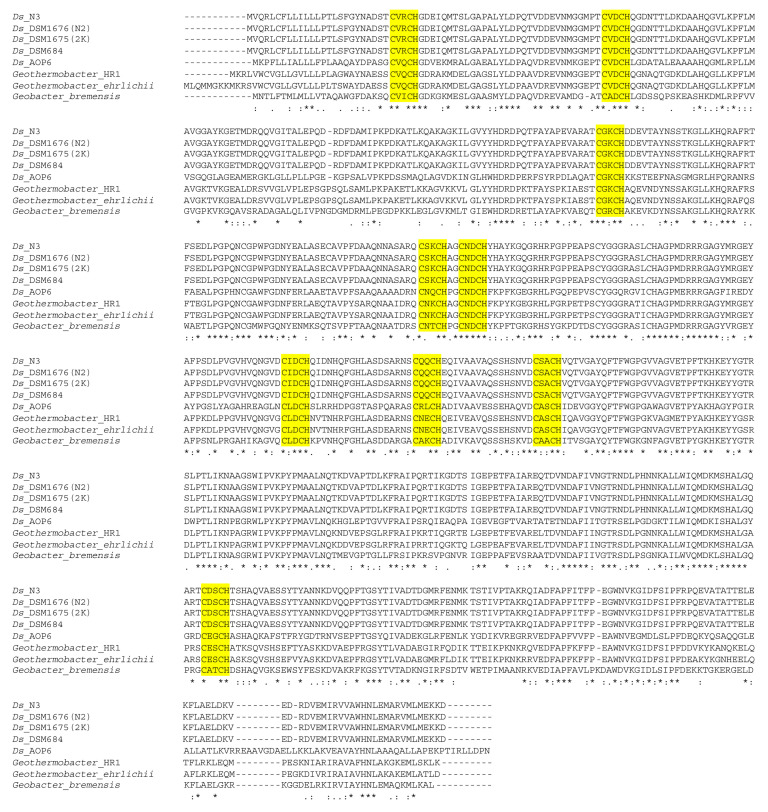
Alignment of the large unique multiheme cytochrome C sequences from *Desulfuromonas* genomes and their closest relatives from *Geothermobacter* and *Geobacter* species. Potential heme-binding sites are marked in yellow. Ds = *Desulfuromonas acetoxidans*.

It has been shown that *Prosthecochloris* could grow by direct interspecies electron transfer from *Geobacter sulfurreducens*, a close relative of *Desulfuromonas* [54]. To elucidate potential unique features in the species that have been shown to form the *Prosthecochloris* syntrophy, we compared the *Geobacter sulfurreducens* genomes of strains PCA and KN400 to the *Desulfuromonas* genomes. This revealed a unique large cytochrome C family protein (624 aa; PGFam_12883110) that is only present in the genomes of *Ds.* DSM1675 (2-K), DSM1676 (N2), N3, 684, AOP6, and the two *Gb. sulfurreducens* strains. An EXPASY-BLASTP search revealed that this is a multiheme cytochrome, with the closest relative being an uncharacterized cytochrome C from *Geothermobacter* sp. HR-1 (67% identity and 80% similarity), and several homologues in other *Geobacter* and *Thermodesulfovibrio* strains, but missing from all the other *Desulfuromonas* strains. The protein contains an N-terminal signal peptide and, as shown in the alignment in Figure 8, at least 9 possible heme-binding sites (identified as CXXCH) were found to be present. The function of this large multiheme cytochrome is currently unknown and the surrounding genes consist of mainly hypothetical genes in all of the *Desulfuromonas* strains where it was found, so it does not appear to be genetically associated with any known specific pathway. The recently published genome of *Ds.* strain AOP6 showed that this strain has genes for a large c-type cytochrome and unique Type IV pili [55], although no further analysis was performed. We now found this to be the case for our four genomes of *Desulfuromonas* as well. Since the *Geobacter* species can produce type IV pili and cytochromes that directly transport electrons through the pili to crystalline Fe(III) and Mn(IV) oxides [47,56], it is possible that this related multiheme cytochrome *c* and the Type IV pili play similar electron transport roles in the *Desulfuromonas* strains that contain these specific proteins. 

## 4. Discussion

It is apparent that all three syntrophic mixtures analyzed, *Cp. ethylica* N2, N3, and 2-K, are virtually the same in both the green and colorless components. That is, the interaction between the two components of the mixtures appears to be highly specific, although no physical connection has been described and the mixtures were isolated from different habitats hundreds of miles apart. However, the finding that both the green component and the colorless component of the three syntrophic mixtures are virtually the same asks for some explanation. It would require to be quite a coincidence considering that the mixtures were isolated from habitats geographically separated by hundreds of miles in Russia and that the type strain of *Desulfuromonas* was isolated from the Antarctic on the far side of the Earth. In support of coincidence, Biebl and Pfennig [18] reported that there is no specificity in the syntrophic growth of *Desulfuromonas* DSM 1675 with green bacteria. As far as growth yield is concerned, *Prosthecochloris* DSM 1685 could be replaced by *Chlorobium* strain DSM 258, and *Desulfuromonas* DSM 1675 could be replaced by *Desulfuromonas* DSM 684^T^ in the syntrophic mixture. However, the doubling times were not measured, which might have shown a difference. A far better explanation of the identity of the three isolates, therefore, is that there is some undetected physical interaction between the two species in the mixtures. 

A cell–cell complex, however fleeting, may require surface recognition proteins as previously postulated for the green bacterial consortia of *C. aggregatum* [13]. In that larger consortium, a single central, nonpigmented, heterotrophic bacterium forms close cell–cell interactions with multiple green-sulfur bacteria (15–40 cells) [13,14]. Even though the metabolic interactions in the *Cp. ethylica* symbiotic relationship are possibly different from the *C. aggregatum* and other large consortia, and are likely driven by sulfur cycling in the case of *Cp. ethylica*, the fact that similar large adhesin/agglutination proteins are detected in *Prosthecochloris ethylica* strains indicates that similar interactions may be formed in this more simple symbiont as those in the larger green sulfur consortia. The fact that this gene cluster appears to be present in other *Prosthecochloris* genomes as well (Figure 4) indicates that the capability to produce large outer membrane adhesin structures might be more widespread among green sulfur bacteria than anticipated.

The presence of unique Tad (tight adhesion) pili genes, only found in the *Prosthecochloris* strains that are known to form a symbiotic relationship, suggests that the structural formation of the cell–cell interactions occurs through specific pili and that large agglutination proteins are expected to help maintain this interaction. Both in the larger *C. aggregatum* and in a similar archaeal consortium with *Nanoarchaeum equitans*, fibers are observed that form periplasmic tubules surrounding the entire cell surface [14,20]. The exact nature of these fibers is still unresolved. However, tight adhesion pili, like the ones we found in the *Prosthecochloris ethylica* genomes, may be involved in the initial formation of the connecting tubules. Possibly, the pili are forming the initial connection before wider periplasmic tubules are established. This process would be similar to the better-known process of how conjugation pili (F pili) help to establish conjugation bridges during the process of DNA conjugation in many bacteria. It is interesting that both the Tad pili and the adhesion protein products described here are best known from studies of bacterial virulence factors. However, based on their presence in other nonpathogenic species, they seem to be more widespread amongst bacteria and are likely involved in many environmentally important syntrophic interactions between bacteria. 

In the *Chloropseudomonas ethylica* syntrophy, the colorless *Ds. acetoxidans* bacterium likely reduces sulfur compounds and returns the resulting H_2_S to the *Prosthecochloris* green sulfur component, as proposed by Biebl and Pfennig [18]. This would allow the green sulfur bacteria to use sulfide for their anoxygenic photosynthesis. Studies have shown that various cytochrome c_7_s can directly reduce metal ions or sulfur, and the *Desulfuromonas* C-551.5 is no exception in that respect [57,58]. Thus, this cytochrome may be responsible for sulfur cycling in the mixtures. In fact, it has previously been shown that *Ds. acetoxidans* cytochrome C-551 is capable of reducing polysulfides and is suggested to be the terminal reductase [59]. It was also shown that another strain of *Prosthecochloris* can grow by direct interspecies electron transfer with *Geobacter sulfurreducens* (a close relative of *Desulfuromonas*) as the electron donor and that they could form a cell–cell complex [54]. These results suggest that the *Desulfuromonas* c_7_ (C-551.5) in the *Chloropseudomonas* mixtures may transfer electrons directly to *Prosthecochloris* without reducing sulfur to sulfide, although that too is a possibility, as shown above. Based on our findings of geopilin-type pili genes and of a larger multiheme cytochrome C gene found exclusively in the genomes of the strains that form these syntrophic mixtures, it is likely that a more complex system of electron transfer through e-pili and multiheme cytochromes is involved, with cytochrome C-551.5 functioning as the terminal sulfur reducing agent in this complex. 

Large multiheme cytochromes with monomeric molecular masses of 50 and 65 kDa (containing 6 and 8 hemes, respectively) were previously observed in *Ds. acetoxidans*, but no sequence data to compare with our current multiheme cytochrome C are available [59]. These multiheme cytochromes were found to cover an extremely wide range of reduction potential, but did not show any hydroxylamine oxidoreductase nor polysulfide reductase activity. Multiheme cytochromes of *Geobacter* and *Desulfuromonas* have been shown to play critical roles in the processing of many metals [57,60], although the monomers were much smaller in size (70–80 residues). A recent review on the role of multiheme cytochromes in anaerobic bacterial respiration [61] reports that multiheme cytochromes with a large molecular ratio of weight/heme ratio (7kDa/heme, or higher) appear to be most common in so-called electroactive organisms that are involved in the reduction of extracellular substrates, such as *Geobacteraceae* and *Shewanellaceae.* The mechanism used by *Geobacter* to transfer electrons onto solid extracellular substrates is still poorly understood, but involves both a pool of periplasmatic cytochromes and several outer membrane multiheme cytochromes. At least some of these form larger polymers [61,62]. The exact electron transfer process is still undetermined, but it is likely a complex multicomponent system where at least some of the multiheme cytochromes have overlapping functions covering the wide range of reduction potentials. By sequence homology alone we cannot state the function of the unique larger multiheme cytochrome C from the *Desulfuromonas acetoxidans* strains, but its high molecular weight/heme ratio (estimated to be 7.5 kDa/heme), N-terminal signal sequence, and unique homology to *Geobacter* large multiheme cytochromes point to a role in extracellular electron transfer. 

**Figure 9:**
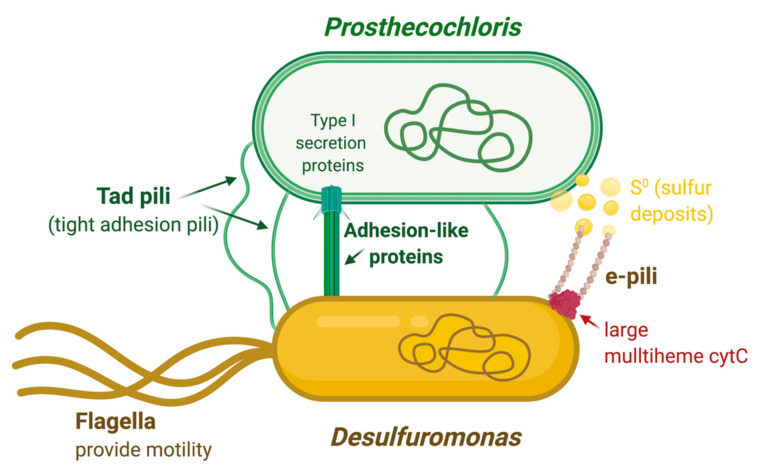
Schematic overview of proposed interactions in the *Chloropseudomonas ethylica* syntrophic mixture. Unique protein structures identified in the genomes are indicated in green for *Prosthecochloris ethylica* and in brown/red for *Desulfuromonas acetoxidans*. Image created in BioRender.com.

Our findings from the simultaneously sequencing and comparison of the genomes in the *Cp. ethylica* syntrophic mixtures, in combination with observations described in related organisms, allow us to propose a basic model by which Tad pili and large agglutination proteins from the green sulfur *Prosthecochloris* are key elements in the formation of the syntrophic complex (Figure 9). This may be in conjunction with the formation of periplasmic tubules that were observed in larger photosynthetic consortia. The formation of larger consortia is dependent on cultivation conditions and is not easily reproduced under laboratory conditions. Although this has not been observed yet in *Cp. ethylica*, given the detection of similar adhesion proteins and pili, it is possible that it could produce similar larger consortia under the right cultivation conditions. Once close cell–cell interactions are formed, the closed sulfur cycle can be established by electron transfer through specialized e-pili and several cytochromes produced by the *Desulfuromonas* component (Figure 9). Further biochemical studies will be needed to determine the exact electron transfer chain and the function of the cytochromes involved, but it is likely that cytochrome C-551.5 plays an essential role in the sulfur reduction. The sulfur metabolism of green sulfur bacteria involves the oxidation of sulfide and the deposition of elemental sulfur globules outside the cells [63]. The e-pili produced by *Desulfuromonas* are therefore expected to be involved in extracellular electron transfer to the sulfur acceptor deposited extracellularly by the green sulfur bacterium (Figure 9). The expression of both specific geopilin and MSHA pili (particularly the major pilin *msh*A gene) in the related organism *Pelobacter carbinolicus* was found to be substantially upregulated during ethanol oxidation, presumably for improved attachment or electron transfer to the extracellular electron acceptor S^0^ [50]. It requires further functional analysis, but the conserved ferredoxin protein directly upstream of the geopilin gene in *Ds. acetoxidans* may potentially be involved in the electron transfer through the e-pili. 

Irrespective of the electron transfer proteins involved, the mutualistic metabolic benefits to each of the components are clear from the sulfur and electron cycling. In addition, the colorless *Desulfuromonas* also contains several genes for flagella production, and, therefore, provides the additional benefit of motility. The potential advantage of motility was previously proposed for the larger *C. aggregatum* consortium [14] and appears to be a part of the *Cp. ethylica* syntrophy as well. None of the green sulfur bacteria observed in these consortia produce flagella and obtaining motility provided by the nonpigmented component is likely to result in a competitive advantage to orient themselves much faster towards light and sulfide gradients in stratified or meromictic lakes.

## Figures and Tables

**Table 1:** Genome characteristics of the *Prosthecochloris* and *Desulfuromonas* genomes used in this study.

Species	Genome Size	GC Content	Contigs	Coverage	CDS	tRNAs	Reference	Genbank Accession #
*Prosthecochloris (N3 mix)*	2.45 Mb	55.1	72	223x	2480	45	this study	JADGII000000000
*Prosthecochloris (N2 mix)*	2.47 Mb	55.1	60	187x	2340	46	this study	JADGIH000000000
*Prosthecochloris (2K, DSM 1685)*	2.44 Mb	55.1	66	123x	2348	45	this study	JABVZQ010000000
*Desulfuromonas (N3 mix)*	3.78 Mb	51.9	121	84x	3557	51	this study	JADGIJ000000000
*Desulfuromonas (N2, DSM 1676)*	3.81 Mb	51.9	52	38x	3567	55	this study	JABWTG010000000
*Desulfuromonas (2K, DSM 1675)*	3.80 Mb	51.9	59	39x	3550	55	this study	JABWTF010000000
*Desulfuromonas DSM 684*	3.83 Mb	51.8	51	N/A	3573	49	unpublished	NZ_AAEW00000000

**Table 2:** Percentage Average Nucleotide Identity (ANIb) of *Prosthecochloris* green bacteria.

*Pr. ethylica* DSM 1685, isolated in pure culture from the *Cp. ethylica* 2K mixture											
**99.91**	*Pr. ethylica* N2 in the *Cp. ethylica* N2 mixture										
**99.95**	**99.93**	*Pr. ethylica* N3 in the *Cp. ethylica* N3 mixture									
**74.5**	**74.7**	**74.6**	*Prosthecochloris* sp HL130								
71	70.9	70.9	70.3	*Prosthecochloris* sp Ty Vent							
72.8	73	72.9	72.2	70.8	*Pr. aestuarii* DSM 271						
69.5	69.5	69.5	69.5	70.5	70.2	*Pr. marina* VI					
69.8	69.9	69.8	69.6	71.1	70.5	**77.2**	*Prosthecochloris* sp A305				
70.3	70.3	70.3	70.5	71.5	71.4	**74.6**	**76.3**	*Pr. phaeobacteroides* BS1			
72.2	72.3	72.3	72	70	71.2	68.9	69	70.4	*Pr. vibrioforme* DSM 260		
72	72.2	72.1	71.8	69.9	71	68.9	69	70.3	**98.3**	*Pr. phaeum* 2401	

Bold means: ANI values above 95% which is the arbitrary cutoff value for species differentiation.

**Table 3:** Percentage average nucleotide identity of some *Desulfuromonas* sulfur-reducing bacteria.

*Ds. acetoxidans* DSM 684^T^ pure culture								
**99.92**	*Ds. acetoxidans* 2K DSM 1675 pure culture							
**99.91**	**99.99**	*Ds. acetoxidans* N2 DSM 1676 pure culture						
**99.86**	**99.92**	**99.93**	*Ds. acetoxidans* in the *Cp. ethylica* N3 mixture					
69.9	69.9	70	70	*Ds. thiophila* DSM 8987				
67.2	67.4	67.4	67.4	68.9	*Ds. acetexigens* null			
67.4	67.5	67.5	67.4	68.7	72.1	*Ds. sp.* WTL		
67.2	67.3	67.3	67.2	69.5	71.5	72.3	*Ds.* sp. DDH 964	

Bold: ANI values above 95% which is the arbitrary cutoff value for species differentiation.

**Table 4:** Overview of some of the PGFams uniquely identified in *Ds*. strains DSM 1675 (2-K), DSM 1676 (N2), N3, and DSM 684 (red clade in Figure 5).

Gene	PGFam	Protein Description		Size (aa.)
PilA	PGF_00056426	Tfp pilus major pilin assembly protein		314
PilZ	PGF_00414431	Type IV pilus assembly protein		121
PilX	PGF_03889320	Type IV fimbrial biogenesis protein		174
	PGF_04686021			204
PilV	PGF_04940117	Type IV fimbrial biogenesis protein		141
	PGF_05800309			126
PilP	PGF_06326322	Type IV fimbrial biogenesis protein		171
PilW	PGF_10370792	Type IV fimbrial biogenesis protein		330
FimT	PGF_10544072	Type IV fimbrial biogenesis protein		143
PglB	PGF_05008343	Pilin glycosylation protein		206
Ferro	PGF_00004340	Ferrodoxin domain protein		260
MshA	PGF_06702067	MSHA pilin protein		169
	PGF_12682451			178
MshD	PGF_01602851	MSHA pilin protein		152
MshI	PGF_05907321	MSHA biogenesis protein		198
MshJ	PGF_02203893	MSHA biogenesis protein		230
MshK	PGF_00018410	MSHA biogenesis protein		119
MshN	PGF_00938251	MSHA biogenesis protein		430
MshP	PGF_01166820	MSHA biogenesis protein		130
CytC	PGF_12883110	Multiheme Cytochrome C family protein		624
